# Tissue-Resident Macrophages in the Stria Vascularis

**DOI:** 10.3389/fneur.2022.818395

**Published:** 2022-02-03

**Authors:** Taku Ito, Natsuko Kurata, Yoko Fukunaga

**Affiliations:** Department of Otorhinolaryngology, Tokyo Medical and Dental University, Tokyo, Japan

**Keywords:** stria vascularis, macrophages, hearing loss, inflammation, activation

## Abstract

Tissue-resident macrophages play an important role in clearance, development, and regulation of metabolism. They also function as sentinel immune cells, initiating inflammatory responses, clearing inflammatory debris, and maintaining homeostatic tissue environment. In the cochlea, the roles of tissue-resident macrophages include maintaining steady-state tissues, immunological defense, and repairing pathological conditions associated with noise, ototoxic drugs, aging, and various pathogens. Perivascular macrophages (PVMs) are a unique subset of tissue-resident macrophages that are closely associated with blood vessels and have unique expression markers in certain tissues. PVMs are found in the inner ear, brain, skin, liver, and retina. The origin of PVMs in the inner ear is unclear, but they are already present during embryonic development. PVMs are members of the blood labyrinth barrier and regulate blood vessel permeability in the stria vascularis, which lies on the lateral wall of the cochlear duct and is crucial for endocochlear potential formation. The cytoplasm of strial PVMs can contain pigment granules that increase in number with age. Strial PVMs are activated by the loss of *Slc26a4* in the cochleae, and they subsequently phagocytose aggregated pigment granules and possibly degenerated intermediate cells. This review summarizes the current knowledge of characteristic features and proposed roles of PVMs in the stria vascularis. We also address macrophage activation and involvement of pigment granules with the loss of *Slc26a4* in the cochleae.

## Introduction

Macrophages are present in almost all body tissues and display location-specific functions and gene expression profiles. These “tissue-resident macrophages” play an important role in the clearance, development, and regulation of metabolism depending on the tissue location and environment (1). They also function as immune sentinels, initiating inflammatory responses, clearing inflammatory debris, and maintaining a homeostatic tissue environment (1). Tissue-resident macrophages were classically believed to originate from the bone marrow via circulating monocytes (2, 3). However, recent studies provide evidence regarding the existence of monocyte-independent macrophage precursors in the yolk sac and fetal liver during developmental stages (1, 4, 5). Presently, it is thought that most adult tissue-resident macrophages are already formed during the embryonic period and persist and maintain themselves locally throughout adulthood via self-renewal.

Macrophages can support angiogenesis (6)— formation or expansion of new blood vessels during development and postnatal life (7, 8). During normal vascular development, macrophages directly interact with, and modulate, the developing vasculature (9, 10). Physical contact between macrophages and growing blood vessels coordinates vascular fusion (9). Perivascular macrophages (PVMs) are a unique subset of tissue-resident macrophages that are closely associated with blood vessels and have unique expression markers in certain tissues. In adult tissues, PVMs are found in the inner ear, brain, skin, liver, and retina. PVMs maintain tight junctions between endothelial cells and limit vessel permeability, phagocytose potential pathogens before they enter tissues from the blood, and restrict inappropriate inflammation. In the brain, PVMs contribute to increased vascular permeability and granulocyte recruitment in the acute phase of stroke (5). Phagocytosis is another role of intracranial PVMs, and is crucial for preserving the cerebrovasculature (11). In the retina, PVMs move along the abluminal surface of the blood vessel endothelium, scavenging potential pathogenic substances present in the perivascular space (12).

Tissue-resident macrophages are present at various locations in the cochlea, including the spiral ganglion, spiral ligament, and stria vascularis. The stria vascularis is located on the lateral wall of the cochlear duct, which is essential for the generation of endocochlear potential and hearing acquisition. In this review, we provide a comprehensive overview of the origins, specifications, possible functions, and activation of tissue-resident macrophages in the stria vascularis. We also relate these with the recent findings of our study in the field of macrophage research and highlight important questions that remain unanswered.

## Source of Tissue Resident Macrophage in Stria Vascularis

During development, Iba1-positive resident macrophages appear in the mouse otocyst as early as embryonic day 10.5, and are distributed in the spiral ganglion and spiral ligament until birth (4, 13). Csf1r signaling promotes the development of Iba1-positive macrophages from the yolk sac during the embryonic period (4). This is confirmed by evidence that they are selectively decreased in ears without Csf1r signaling. In contrast, CD11b-positive cells were observed on the cochlear mesenchyme at embryonic day 14.5, which are deemed to be resident macrophages originating from the fetal liver. They reside in the mesenchyme of the cochlear modiolus or the intraluminal surface of the perilymphatic space, and most are distinct from Iba1-positive resident macrophages. Macrophages in the stria vascularis can be observed only after birth, increase during the neonatal stages, and are Iba1-positive (4). They are always distributed close to blood vessels in the stria vascularis. The unique timing of appearance and location suggest that the origin of resident macrophages in the stria vascularis may be different from that of macrophages at other sites of the cochleae.

In the adult inner ear, bone marrow-derived resident macrophages are present in a steady state in the spiral ligament, the acoustic nerve and stria vascularis (14, 15). They are an interchangeable and migratory population that is replenished by the recruitment of blood-borne monocytes (16). Tissue-resident macrophages in the stria vascularis of the adult cochleae are positive for F4/80, CD68, Iba1, and CD11b (16–18) and are mainly localized around blood vessels, suggesting that almost all of them are PVMs. Some researchers propose that PVMs in the stria vascularis originate from cochlear melanocytes derived from the neural crest that have migrated to the stria vascularis (19–21). However, the cell populations of neural crest origin are distinct from those of CD68-positive cells in the embryonic cochlea (4). Considering the different developmental lineages and phenotypes of macrophages and melanocytes, PVMs are unlikely to be derived from the neural crest (22). Taken together, the data demonstrate that strial PVMs change their characteristic expression markers during postnatal development, and are renewed or replaced by blood-borne monocytes in the adult inner ear.

## Tissue Resident Macrophages as Members of the Blood Labyrinth Barrier in Stria Vascularis

There are three separate fluid compartments in the cochlea: the endolymphatic, perilymphatic, and intrastrial spaces. Each compartment is morphologically and electrically separated by tight junctions. Tight junctions are directly involved in intercellular sealing. Leakage of solutes through the paracellular pathway to the intrastrial space from other compartments is prevented by various molecules, including claudin-1, claudin-3, and claudin-4 in the marginal cells on the medial side (23), and claudin-11 in the basal cells on the lateral side (24). Intrastrial fluid is a unique extracellular solution with a highly positive potential, similar to that of the endolymph, but a low potassium concentration, as observed in the perilymph (25). The intrastrial space is also isolated by tight junctions of vascular endothelial cells from the intravascular lumen. This isolation is referred to as the blood-labyrinth barrier. The barrier consists of several tight junctions and adherent junction proteins, including ZO-1, occludin, and VE-cadherin (20, 21). Endothelial cells are sheathed by a dense basement membrane shared with pericytes. Perivascular macrophages further cover the capillary surface (21, 26). PVMs in the stria vascularis are composed of long-branching processes and a small cellular body. The cell body and its branches are always located adjacent to blood vessels (**Figures 2A,C**). This characteristic shape is similar to that of ramified microglia in the central nervous system (CNS). Iba-1 positive macrophages with similar form have also been reported in the human cochlea (26, 27).

Previous studies suggest that these PVMs are integrated into the structure of cochlear vessels and maintain the integrity of the blood-labyrinth barrier between the blood and intrastrial space (20). The integrity of this blood-strial barrier is critical for solute homeostasis, maintenance of high endocochlear potential, and normal hearing. Collapse of this barrier integrity results in a reduction in endocochlear potential (28–30). A previous study demonstrated that the elimination of PVMs results in leaky capillaries and a reduction in endocochlear potential (20). Hirose et al. (22), in contrast, reported that PVM depletion does not alter the baseline permeability of cochlear vessels, and PVMs in the stria vascularis play an active role in opening the blood strial barrier, depending on the tissue environment (22). This discrepancy might be due to the method of assessing barrier function, genetic background of the tested mice, or other factors (22). In this regard, the latter idea is more consistent with a previous report that intraperitoneally injected tracers are incorporated into macrophages in the stria vascularis (16).

## Tissue Resident Macrophages Likely Phagocytose Melanin Pigments in the Stria Vascularis

Melanin is synthesized in melanocytes and plays diverse roles and functions in various organs. Intermediate cells are melanocyte-like cells, presumably derived from the neural crest, that normally synthesize melanin pigment. Melanin in the stria vascularis is considered to play protective roles against noise, ototoxins, and aging. The protective properties of melanin may be derived from its ability to bind cations and metals and scavenge free radicals (31–33). Once synthesized by strial intermediate cells, melanin is exported to the intrastrial space in melanosomes, where it may remain or be taken up by marginal or basal cells (34). The majority of melanin might be taken up by marginal cells, since they are the most vulnerable cell population in the stria vascularis (35, 36). Noise, ototoxins, and aging increase melanosome extrusion by intermediate cells; however, it is currently unclear how melanin is transported, digested, decomposed, or removed (33).

**Figure 1:**
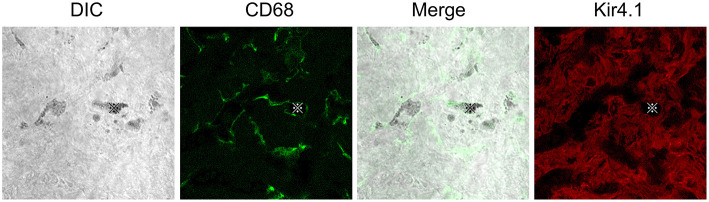
Distributions of CD68 or Kir4.1 positive cells in the stria vascularis. Whole-mount preparation of the stria vascularis stained with anti-*CD68* (green) or *Kir4.1* (red) antibodies to label macrophages or intermediate cells. DIC images show pigmentations phagocytized by *CD68*-positive macrophages. The *CD68* expression pattern is entirely different from that of *Kir4.1*, suggesting that *CD68*-positive macrophages are distinct from *Kir4.1*-positive intermediate cells *in vivo*. Reproduced from Figure 9, Ito et al. (17).

**Figure 2:**
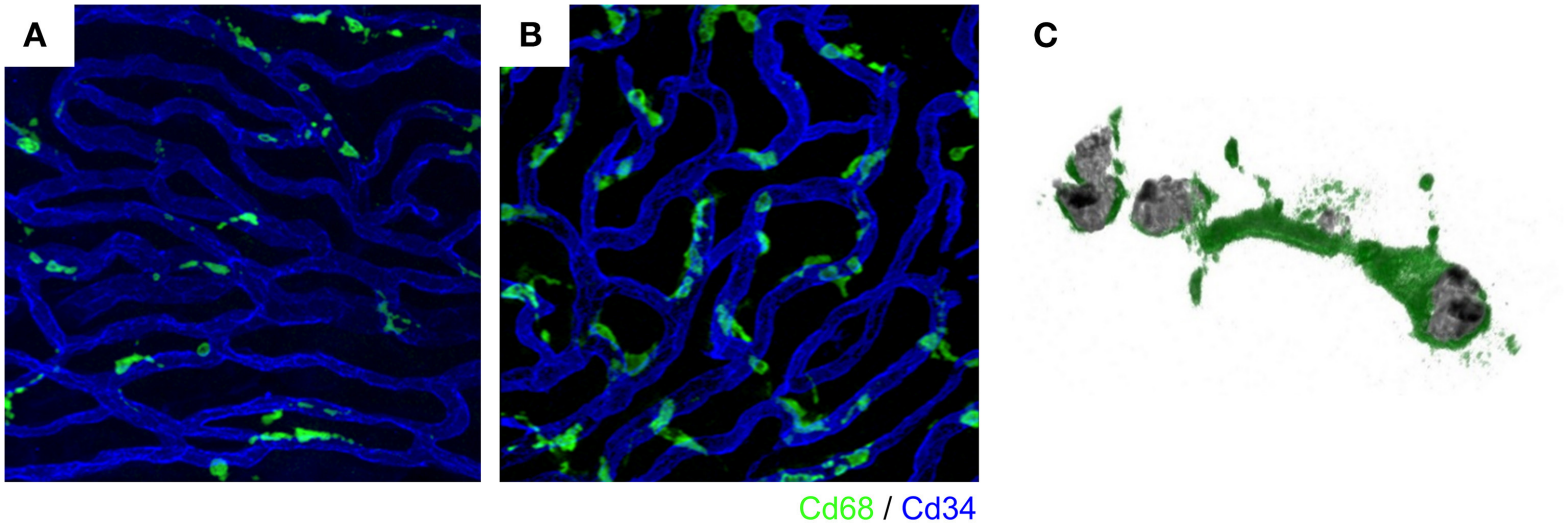
Intravascular vessels and macrophages. A rich vascular network develops within the stria vascularis and perivascular macrophages exist close to the vessels. In the steady state, stria vascularis, macrophages have a ramified cell morphology with multiple finely branched projections on a small cell body **(A)**. Activated macrophages change to an amoeboid form in the ears of *Slc26a4*-null mice, displaying larger somata and shorter, coarser cytoplasmic processes **(B)**. Activated macrophages with phagocytosed aggregated pigment granules **(C)**. The vasculature and macrophages were labeled with anti-CD34 (blue) and anti-CD68 (green) antibodies, respectively **(A,B)**. Reproduced from Figure 7, Ito et al. (17, 39).

Macrophages contribute to tissue homeostasis by phagocytosing cellular debris, foreign agents, and apoptotic cells. They also ingest self-derived particulates, such as melanin. Macrophages containing melanin granules are observed in normal human skin and are referred to as “melanophages” (37, 38). Melanin granules were also observed in the PVMs of the mouse stria vascularis. Some reports propose the term “perivascular macrophage-like melanocyte (PVM/Ms)” to describe such macrophages, including melanin pigments in the stria vascularis (19, 20). PVM/Ms are referred to as a hybrid cell type with characteristics of both macrophage and melanocyte surface markers, including F4/80, CD68, CD11b, and Kir 4.1. Kir 4.1, encoded by the *Kcnj10* gene, is widely accepted as an intermediate cell marker (20). However, CD68-positive cells containing pigment granules appeared distinct from Kir4.1-positive cells *in vivo* in our previous study (17) (Figure 1). Instead, they appear to phagocytose damaged intermediate cells, marginal cells, or the melanin granule itself in the intrastrial space (Figure 2C) (39). The involvement of PVMs with melanin granules, intermediate cells, and marginal cells in the stria vascularis remains unclear; however, some types of tissue-resident macrophages function to not only remove waste products but also execute cell death by phagocytosing stressed-but-viable cells (40, 41). PVMs in the stria vascularis may accelerate the clearance of apoptotic cells. Lastly, an excess amount of pigment granules may cause damaged intermediate and marginal cells to be engulfed before they are repaired.

## Increment and Activation of Tissue Resident Macrophage in Pendred Syndrome

**Figure 3:**
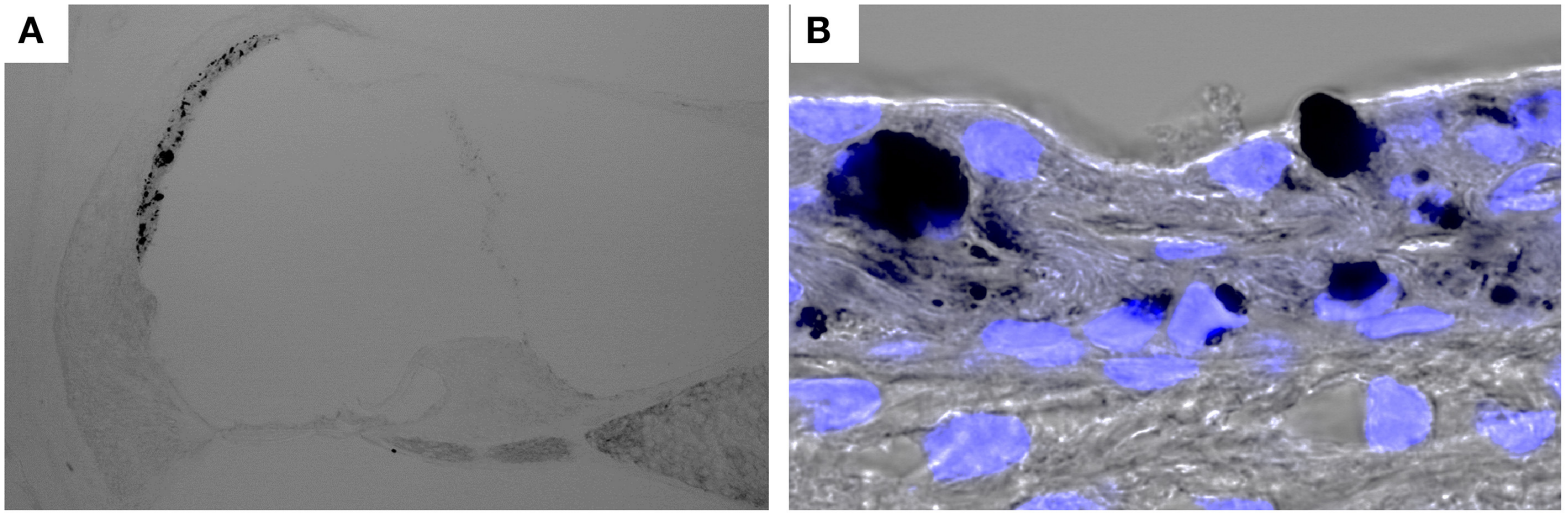
Hyperpigmentation in a mouse model of Pendred syndrome. *Slc26a4*-insufficient cochlear sections were stained with DAPI. Hyperpigmentation is solely observed in the stria vascularis **(A)** and pigment granules are localized in the basal, intermediate and marginal cell layers **(B)**. *Slc26a4* expression is controlled by doxycycline in *Slc26a4*-insufficient (Tg[E];Tg[R];*Slc*26*a*4^Δ/Δ^) mice (46).

Pendred syndrome is characterized by autosomal recessive inheritance of mutations to the *SLC26A4* gene, goiter, hearing loss, and enlargement of the vestibular aqueduct. The stria vascularis is atrophied and hyperpigmented in the ears of *Slc26a4*-null mice, a model of Pendred syndrome (42). Hyperpigmentation was observed solely in the stria vascularis across all cochlear turns, and pigment granules were present throughout the marginal, intermediate, and basal cell layers (Figure 3). Additionally, significant macrophage proliferation and activation have been observed in the stria vascularis (18). Macrophages in the stria vascularis have multiple projections and a slim cell body, and most cells exist adjacent to blood vessels in the steady state. In contrast, activated macrophages shift to an amoeboid morphology with larger somata and shorter, coarser cytoplasmic processes in the ears of *Slc26a4*-null mice (Figures 2B,C). Despite such an apparent switch, the cells are not observed migrating away from the vascular periphery, and always have a part of their cells in contact with the vessel wall. We demonstrated that macrophage incrementation was correlated with the severity of hearing loss, suggesting that macrophage activity affects hearing by influencing the function of the stria vascularis (17).

Macrophage proliferation and activation in the ears of *Slc26a4*-null mice are not accompanied by upregulation of acute inflammatory markers or neutrophil invasion (18). In the CNS, microglia can be activated by a variety of factors, including pro-inflammatory cytokines, cell necrosis factors, lipopolysaccharides, and changes in extracellular potassium, which are indicative of ruptured cells. However, the specific signals underlying macrophage activation in the inner ear remain unclear. They may be associated with oxidative and nitrative stress signals, which are elevated in the ears of *Slc26a4*-null mice (43), Hyperpigmentation can also activate perivascular macrophages. The molecular and biological pathways of perivascular macrophage activation and the role of hyperpigmentation remain unclear. Activated amoeboid microglia in the CNS phagocytose foreign materials, extracellular debris, and apoptotic cells, and then display the resulting immunomolecules for T-cell activation (40, 44). Similar pathogenesis may be present in the stria vascularis of *Slc26a4*-null mice. Thus, the histological hallmarks of these pathogenic changes may provide clues for the development of novel therapeutic medications for the treatment of hearing loss.

## Macrophage and Autoinflammation of the Inner Ear

Autoinflammatory diseases are caused by dysfunction of the innate immune system. They are characterized by either periodic or chronic systemic inflammation, usually without the involvement of adaptive immunity, and some patients exhibit sensorineural hearing loss. The common pathophysiological feature of autoinflammatory diseases is the increased production of interleukin-1 (IL-1), which is predominantly produced by activated macrophages as a pro-protein, pro-IL-1β. Cochlear resident macrophages secrete pro-IL-1β through NLRP3 inflammasome activation (45). Knowledge of autoinflammatory inner ear diseases is limited; therefore, it remains unclear how strial macrophages would play a role in causing hearing loss in these diseases. Further investigation to identify the characteristics of perivascular resident macrophages may potentially shed light on the pathophysiology of hearing loss with unknown cause.

## Conclusion

**Figure 4:**
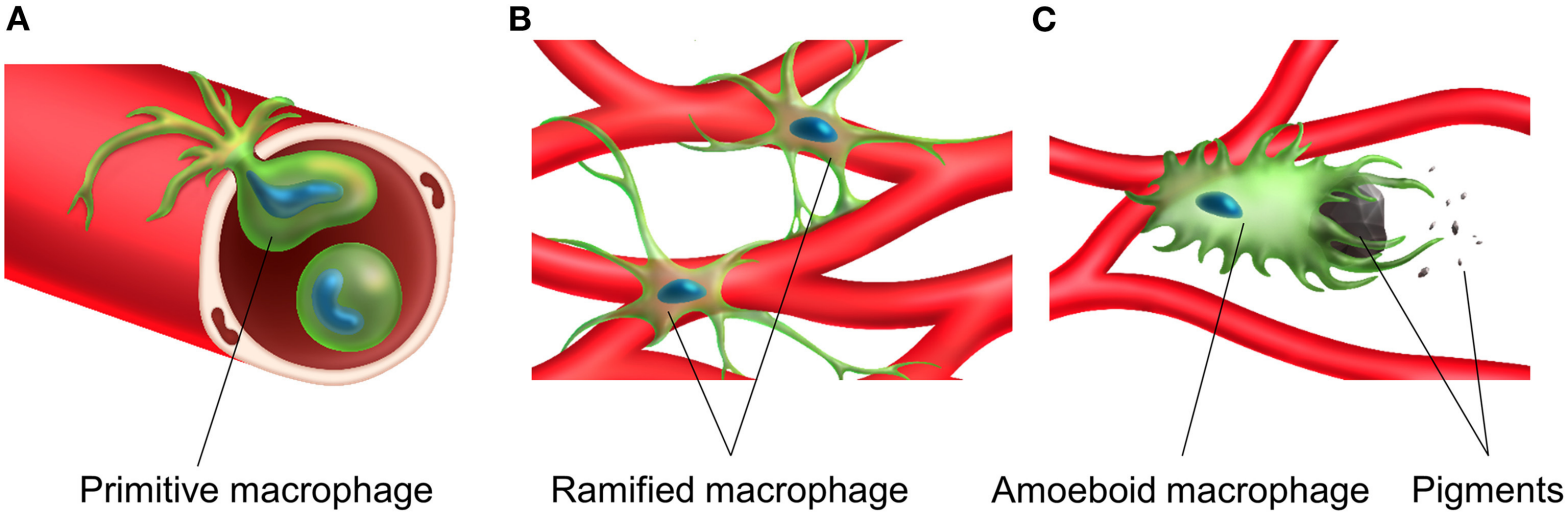
Transportation, distribution and activation of tissue resident macrophage in the stria vascularis. A primitive macrophage travels in the bloodstream **(A)** and is distributed on the surface of blood vessels in the stria vascularis. Macrophages exist near the rich vascular network and have a ramified cell morphology with multiple finely branched projections on a small cell body in the steady-state stria vascularis **(B)**. Macrophage activation is associated with melanin and activated macrophages appear to phagocytose aggregated pigment granules **(C)**.

Tissue-resident macrophages in the stria vascularis are observed only during the postnatal period and are always distributed close to blood vessels. Their possible functions and roles include adjustment of the blood-labyrinth barrier integrity and maintenance of homeostasis in the intrastrial space. The morphology of tissue-resident macrophages in the stria vascularis changes upon activation in the presence of melanin pigments, which might accelerate the degeneration of the stria vascularis in the ears of *Slc26a4*-null mice (Figure 4).

## Author Contributions

TI and NK wrote the manuscript. YF provided critical feedback. All of the authors should have read and critically reviewed the manuscript. All authors contributed to the article and approved the submitted version.

## Conflict of Interest

The authors declare that the research was conducted in the absence of any commercial or financial relationships that could be construed as a potential conflict of interest.

## Publisher's Note

All claims expressed in this article are solely those of the authors and do not necessarily represent those of their affiliated organizations, or those of the publisher, the editors and the reviewers. Any product that may be evaluated in this article, or claim that may be made by its manufacturer, is not guaranteed or endorsed by the publisher.
